# Effect of Roux-en-Y Bariatric Bypass Surgery on Subclinical Atherosclerosis and Oxidative Stress Markers in Leukocytes of Obese Patients: A One-Year Follow-Up Study

**DOI:** 10.3390/antiox9080734

**Published:** 2020-08-11

**Authors:** Zaida Abad-Jiménez, Sandra López-Domènech, Segundo Ángel Gómez-Abril, Dolores Periañez-Gómez, Aranzazu M. de Marañón, Celia Bañuls, Carlos Morillas, Víctor M. Víctor, Milagros Rocha

**Affiliations:** 1 Department of Endocrinology and Nutrition, University Hospital Doctor Peset, Foundation for the Promotion of Health and Biomedical Research in the Valencian Region (FISABIO), 46017 Valencia, Spain; zaiaji@alumni.uv.es (Z.A.-J.); sandra.lopez@uv.es (S.L.-D.); amardema@alumni.uv.es (A.M.d.M.); celia.banuls@uv.es (C.B.); carlos.morillas@uv.es (C.M.); 2 Department of General and Digestive System Surgery, University Hospital Doctor Peset, Foundation for the Promotion of Health and Biomedical Research in the Valencian Region (FISABIO), 46017 Valencia, Spain; gomez_seg@gva.es (S.Á.G.-A.); perianyez_dol@gva.es (D.P.-G.); 3 Department of Surgery, Faculty of Medicine and Dentistry, University of Valencia, Av Blasco Ibáñez 13, 46010 Valencia, Spain; 4 CIBERehd-Department of Pharmacology, University of Valencia, Av Blasco Ibáñez 13, 46010 Valencia, Spain; 5 Department of Physiology, Faculty of Medicine and Dentistry, University of Valencia, 46010 Valencia, Spain

**Keywords:** obesity, bariatric surgery, oxidative stress, atherosclerosis, leukocyte-endothelium interactions

## Abstract

Little is known about the mechanisms underlying the cardioprotective effect of Roux en-Y gastric bypass (RYGB) surgery. Therefore, the aim of the present study was to investigate whether weight loss associated with RYGB improves the oxidative status of leukocytes and ameliorates subclinical atherosclerotic markers. This is an interventional study of 57 obese subjects who underwent RYGB surgery. We determined biochemical parameters and qualitative analysis of cholesterol, leukocyte and systemic oxidative stress markers —superoxide production, glutathione peroxidase 1 (GPX1), superoxide dismutase (SOD) activity and protein carbonylation—, soluble cellular adhesion molecules —sICAM-1 and sP-selectin—, myeloperoxidase (MPO) and leukocyte-endothelium cell interactions—rolling flux, velocity and adhesion. RYGB induced an improvement in metabolic parameters, including hsCRP and leukocyte count (*p* < 0.001, for both). This was associated with an amelioration in oxidative stress, since superoxide production and protein carbonylation were reduced (*p* < 0.05 and *p* < 0.01, respectively) and antioxidant systems were enhanced (GPX1; *p* < 0.05 and SOD; *p* < 0.01). In addition, a significant reduction of the following parameters was observed one year after RYGB: MPO and sICAM (*p* < 0.05, for both), sPselectin and pattern B of LDL particles (*p* < 0.001, for both), and rolling flux and adhesion of leukocytes (*p* < 0.05 and *p* < 0.01, respectively). Our results suggest that patients undergoing RYGB benefit from an amelioration of the prooxidant status of leukocytes, metabolic outcomes, and subclinical markers of atherosclerosis.

## 1. Introduction

Obesity is an endocrine disease with an important inflammatory component [1] that underlies the development of clinical complications such as type 2 diabetes (T2D), arterial hypertension, dyslipidemia and metabolic syndrome, all of which are considered major risk factors for cardiovascular disease (CVD) [2]. Indeed, excess weight and, particularly, accumulation of abdominal fat have also been epidemiologically associated with increased cardiovascular morbidity and mortality [3,4]. Vascular health is impaired in several ways during obesity, including arterial stiffening, endothelial dysfunction and atherosclerosis, which are primary phases in the development of major cardiovascular complications including stroke and coronary diseases. In this context, proinflammatory status, insulin resistance (IR), and oxidative stress are recognized as major inducers of vascular damage and endothelial dysfunction [5,6,7].

Oxidative stress is a hallmark of obesity caused by undermined antioxidant capacity in conjunction with increased levels of reactive oxygen species (ROS) production. In this sense, excess ROS is detected in adipose tissue and immune cells of obese patients and is closely related with alterations in mitochondrial function due to excess nutrient supply [8,9]. Previous studies have reported an impairment of the main antioxidant systems in morbid obesity, including superoxide dismutase (SOD), catalase and glutathione peroxidase (GPX) [10,11], which are considered the front line of enzymatic ROS scavenging. The resulting accumulation of ROS leads to oxidation of macromolecules including lipids, proteins and DNA, eventually affecting cellular homeostasis and function. In particular, oxidizing species cause injury in the vascular wall by interrupting NO bioavailability and oxidizing LDL particles [7].

At the onset of the atherosclerotic process, the exposure of the intimal endothelium to damaging stimuli such as circulating proinflammatory cytokines, activated leukocytes and oxidized LDL (oxLDL) particles triggers the activation of endothelial cells following leukocyte recruitment and transmigration across the endothelial barrier, a process initiated by selectin-dependent adhesion molecules (E-selectin and P-selectin) and mediated by vascular cellular adhesion molecules (VCAM) and intercellular adhesion molecules (ICAM) [12,13]. Once in the subendothelial space, leukocytes scavenge oxLDL particles and transform into foam cells, largely perpetuating the oxidative and inflammatory response and contributing to vascular remodelling. In this regard, our group and others have reported that circulating leukocytes of patients with IR-disorders including obesity, T2D, and polycystic ovary syndrome (PCOS) are in a proinflammatory and prooxidant state, and are associated with the extent of the vascular injury [8,14,15,16]. This accumulating evidence supports a relevant role of immune cells in the pathogenesis of atherosclerosis.

Bariatric surgery has become the most effective therapeutic approach for the treatment of obesity. In particular, the Roux-en-Y gastric bypass (RYGB) offers consistent short- and long-term effects on weight loss maintenance and overall resolution of obesity-associated metabolic comorbidities, resulting in reduced total mortality and incidence of cardiovascular events [17,18]. Accumulating evidence suggests an improvement of vascular function, systemic oxidative stress, and inflammation after bariatric surgery [19,20]. However, little is known about the modulation of immune cell response after surgery-mediated weight loss and the role it plays in the cardioprotective effect of the intervention. In the present study, we aimed to explore the effect of weight loss induced by RYGB surgery on the activation and oxidative status of leukocytes and the endothelial dysfunction associated to obesity. Hence, the primary end-point was to evaluate potential changes in leukocyte-endothelial cell interactions one year after the RYGB. Secondly, we aimed to clarify whether the intervention was associated with changes in subclinical mechanisms of atherosclerosis, including systemic and intracellular oxidative stress, inflammation, and atherogenic dyslipidemia.

## 2. Materials and Methods

### 2.1. Study Population

For this interventional study, a cohort of fifty-seven obese patients with body mass index (BMI) ≥ 35 kg/m^2^ and scheduled for RYGB surgery were recruited between January 2014 and September 2019 from the Outpatient’s Clinic of the Department of Endocrinology and Nutrition, and the Department of General and Digestive System Surgery of the University Hospital Dr. Peset (Valencia, Spain). The study protocol was approved by the hospital’s Human Ethics Committee (code 96/16) and conducted according to the guidelines of the Declaration of Helsinki. All the participants were informed about the objective and methodology of the study and signed a written informed consent.

The inclusion criteria were age 18–65 years, BMI > 40 or > 35 kg/m^2^ with comorbidities, and assignment of RYGB surgery. Exclusion criteria were pregnancy or lactation, active infectious disease, thromboembolism, stroke or documented history of CVD, severe disease including malignancies, severe renal or hepatic disease, drug abuse, chronic inflammatory disease, and secondary obesity (hypothyroidism, Cushing’s syndrome).

All the patients were examined and the study variables recorded at baseline and 12 months after the surgical intervention.

### 2.2. Clinical and Biochemical Determinations

Anthropometric measurements including weight, height, systolic and diastolic blood pressure, and waist circumference were obtained during a physical exploration by means of electronic scales, stadiometer, sphygmomanometer, and metric measuring tape, respectively. BMI was calculated by dividing weight by the square of height. The percentage of excess weight loss (EWL) was calculated with the formula [(preoperative weight−current weight)/(preoperative weight − ideal weight (considering BMI = 25 kg/m^2^))] × 100.

Blood samples were collected from the antecubital vein in fasting conditions at 8:00–9:30 a.m. at baseline and one year after RYGB surgery. Biochemical determinations were performed at the Hospital’s Clinical Analysis Service as follows: Glucose, total cholesterol (TC), and triglycerides (TG) serum levels were determined by the enzymatic assay; HDL cholesterol (HDLc) concentration was measured using a Beckman LX20 analyser (Beckman Coulter Inc., Brea, CA, USA); and LDL cholesterol (LDLc) was calculated by Friedewald’s formula when circulating levels of TG did not exceed 300 mg/dL. The percentage of glycated haemoglobin (HbA1c) was obtained with a glycohaemoglobin analyser (Arkray Inc., Kyoto, Japan). Insulin was determined by an immunochemiluminescence assay and the Homeostatic Model Assessment for Insulin Resistance index (HOMA-IR) calculated with the formula ([fasting insulin (μUI/mL) × fasting glucose (mg/dL)]/405). Systemic levels of high sensitivity C-reactive protein (hsCRP) and C3 fraction of the complement (C3c) were analysed using an immunonephelometric assay (Behring Nephelometer II, Dade Behring, Inc., Newark, DE, USA) with an intra-assay coefficient of variation < 5.5%. Total leukocytes were determined in a COULTER^®^ LH 500 hematology blood analyser (Beckman Coulter Inc., Brea, CA, USA). The remaining serum aliquots were immediately stored at –80 °C for subsequent analysis.

### 2.3. Evaluation of Cellular Adhesion Molecules (CAMs) and Myeloperoxidase (MPO)

Levels of soluble CAMs—sICAM-1 and sP-selectin— and MPO were measured in serum with a Luminex 200 analyser system (Luminex Corporation, Austin, TX, USA) following the Milliplex^®^ MAP Kit manufacturer’s procedure (Millipore Corporation, Billerica, MA, USA). All samples were analysed in duplicate. For all determinations, the intra-serial and inter-serial variation coefficients were <5.0% and <15.0%, respectively.

### 2.4. SOD Activity Assay and Carbonylation of Serum Protein

Activity of serum SOD was evaluated with a commercial kit (Cayman Chemical, Ann Arbor, MI, USA) and the amounts of carbonyl groups in serum proteins were determined with the OxiSelect^TM^ Protein Carbonyl ELISA Kit (Cell Biolabs, Inc., San Diego, CA, USA) according to the manufacturer’s protocol.

### 2.5. Isolation of Leukocytes from Blood Samples

Blood samples from BD Vacutainer^®^ citrated tubes were mixed and incubated with dextran 3% for 45 min at room temperature (RT) and the resulting supernatant was then layered over Ficoll-Hypaque (GE Healthcare, Uppsala, Sweden) and centrifuged at 650× *g* for 25 min at RT. After lysing the remaining erythrocytes with a specific lysis buffer (Sigma-Aldrich, Inc., St. Louis, MO, USA) the pellet was washed and resuspended in HBSS (Capricorn Scientific, Ebsdorfergrund, Germany).

### 2.6. Fluorescence Imaging of Superoxide Production

Determination of superoxide production was assessed by fluorometry using an IX81 Olympus fluorescence microscope coupled with the static cytometry software ScanR version 2.03.2 (Olympus, Hamburg, Germany). Leukocytes were seeded in a 48-well plaque and incubated for 30 min at 37 °C with a Dihydroethidium (DHE) probe for intracellular superoxide determination and with Hoechst 33342 to visualize cell nuclei. Both fluorescent dyes were purchased from Life Technologies (Thermo Fisher Scientific, Waltham, MA, USA).

### 2.7. Western Blotting

For protein extraction leukocytes were lysed on ice for 15 min with a cell lysis buffer (20 mM HEPES pH 7.5, 400 mM NaCl, 20% glycerol, 0.1 mM EDTA, 10 µM Na_2_MoO_4_, 0.5% NP-40, 1 mM dithiothreitol) in the presence of a protease inhibitor mix (10 mM NaF, 1 mM NaVO_3_, 10 mM PNP, 10 mM β-glycerolphosphate). The protein concentration was estimated with a BCA protein assay kit (Thermo Fisher Scientific, Waltham, MA, USA). Twenty five μg of protein was resolved by electrophoresis in a SDS-polyacrylamide gel and then transferred onto a nitrocellulose membrane. The membranes were blocked for 1 h with 5% skimmed milk in TBS-T and then incubated overnight at 4 °C with the primary antibodies anti-GPX1 (Thermo Fisher Scientific, Waltham, MA, USA) and anti-Actin (Sigma-Aldrich, Inc., St. Louis, MO, USA). The chemiluminescence signal was detected with the ECL plus reagent (GE Healthcare, Little Chalfont, UK) following a proper binding step with the HRP-goat anti-rabbit secondary antibody (Millipore Iberica, Madrid, Spain). The Fusion FX5 Acquisition System permitted visualization and the software Bio1D version 15.03a (Vilbert Lourmat, Marne-la-Vallée, France) was employed to quantify the signal by densiometry.

### 2.8. Dynamic Flow-Chamber-Based Adhesion Assay

To evaluate the interaction between immune cells and the endothelium an in vitro model of adhesion assay was used. This model is based on the use of a dynamic parallel-plate flow chamber coupled to an inverted microscope (Nikon Eclipse TE 2000-S, Amstelveen, The Netherlands) connected to a video recorder camera (Sony Exwave HAD, Koeln, Germany). The chamber was assembled with a coverslip of confluent Human Umbilical Vein Endothelial Cells (HUVEC). Endothelial cells had been previously isolated from umbilical cords by collagenase digestion (1 mg/mL in PBS for 17 min) and then cultured over fibronectin-coated plastic small petri dishes in a complete EGM-2 medium (Lonza, Basel, Switzerland). After assembling the chamber, a suspension of 1 million leukocytes in 1 mL of a RPMI medium (Gibco; Thermo Fisher Scientific, Waltham, MA, USA) was drawn across the HUVEC monolayer at 0.36 mL/min while a 5 × 25 mm portion of the cell culture was recorded for 5 min. During the video analysis the following parameters were evaluated: Leukocyte rolling flux was calculated by counting the number of leukocytes rolling over 100 μm^2^ of HUVEC in 1 min; rolling velocity was measured as the mean time it took 20 consecutive leukocytes to move along 100 μm of the endothelial monolayer; and adhesion was evaluated by counting the number of leukocytes maintaining a firm contact with endothelium for 30 s.

### 2.9. LDL and HDL Subfractions

LDLc and HDLc subfractions were identified using the Quantimetrix Lipoprint^®^ system and quantified by the computerized method of the Quantimetrix Lipoprint^®^ system (Quantimetrix Corporation, Redondo Beach, CA, USA) and NIH program version 1.62 (NIH, Bethesda, MD, USA). The Liposure^®^ kit (Quantimetrix Corporation, Redondo Beach, CA, USA) was used for quality control. The LDL electrophoretic profile showed three patterns: Pattern A (cut-off diameter ≥ 268 Å) with predominance of large and buoyant LDL particles; Intermediate Pattern (cut-off diameter > 265 and ≤ 268 Å); and Pattern B (cut-off diameter ≤ 265 Å), with a predominance of small and dense LDL (sdLDL) particles. The qualitative HDL size analysis divided HDL into 10 subfractions: 1–3 represented large HDL particles, 4–7 indicated medium HDL particles, and 8–10 represented small HDL particles.

### 2.10. Statistical Analysis

This study was primarily designed to achieve a power of 80% and to detect differences in relation to the primary efficacy criterion—i.e., leukocyte adhesion ≥ 5 cells/mm^2^—assuming a common SD of eight units. Under these considerations, a minimum of 21 subjects were required. SPSS 20.0 (IBM SPSS Statistic, Chicago, IL, USA) was used to carry out the statistical analysis. Parametric data are expressed as the mean ± SD or mean + SE and non-parametric data as the median and interquartile range (25% and 75% percentile). Differences between parametric variables were compared with a paired Student’s *t*-test and a Wilcoxon test was used for comparisons of non-parametric data. An *X*^2^ test was used to compare proportions. Statistically significant differences were considered when *p* < 0.05.

## 3. Results

This study evaluated a total of 57 obese patients (7 men and 50 women) undergoing RYGB with an average BMI of 39.8 and an age of 45.4 years old at the beginning of the study. Anthropometric and metabolic parameters of the study cohort before and after the intervention are shown in Table 1.

One year after RYGB patient EWL was 80.4% and their BMI and waist circumference decreased significantly (*p* < 0.001), indicating the efficacy of RYGB in terms of body weight reduction. In addition, patients experienced a drop in systolic and diastolic blood pressure (*p* < 0.001 and *p* < 0.01, respectively) and an improvement of parameters of glucose metabolism, including fasting glucose levels, insulin, HOMA-IR, and HbA1c with respect to the basal condition (*p* < 0.001). The lipid profile was improved by rising HDLc levels (*p* < 0.001) and decreasing levels of TG (*p* < 0.001), TC (*p* < 0.001), and LDLc (*p* < 0.001). Furthermore, there was a reduction in blood leukocyte count (*p* < 0.001) and a significant decrease in levels of the acute phase inflammation reactants hsCRP and C3c (*p* < 0.001), thus suggesting an amelioration of the systemic inflammatory response. Following these clinical changes, the prevalence of metabolic comorbidities associated with obesity—hypertension, hyperlipidemia, and T2D—within the study population fell from 37%, 23%, and 30%, respectively at the beginning of the study to 16%, 9%, and 4%, respectively one year after the surgical intervention, thus confirming the successful remission of obesity-associated metabolic diseases mediated by RYGB.

### 3.1. Systemic and Leukocyte Oxidative Stress Parameters

**Figure 1:**
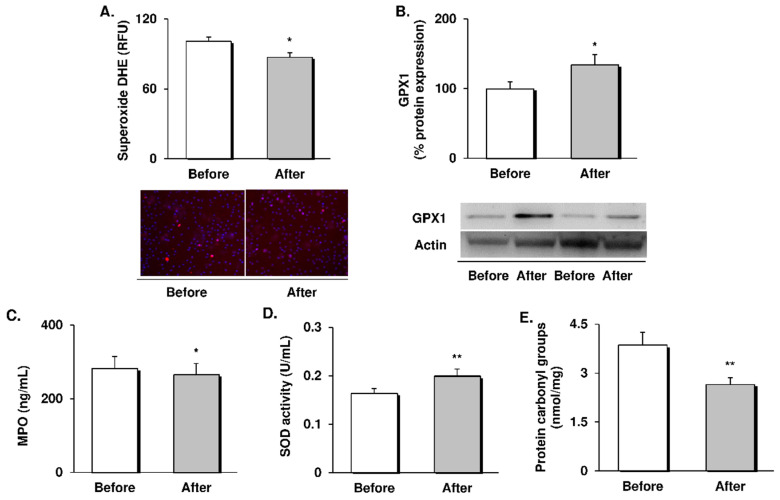
Evaluation of prooxidant/antioxidant responses and oxidative stress markers in obese patients before and 12 months after RYGB intervention. (**A**) Superoxide production in leukocytes, measured as arbitrary units of DHE fluorescence and their representative fluorescence microscopy 100× images (*n* = 17). (**B**) Levels of GPX1 protein expression in leukocytes and representative Western blot images (*n* = 20). (**C**) MPO levels (*n* = 46) and (**D**) SOD activity in serum. (**E**) Carbonyl groups in serum proteins (*n* = 14). Data are represented as the mean + SE. * *p* < 0.05 ** *p* < 0.01 when compared using a paired Student’s t-test. RYGB: Roux-en-Y gastric bypass; DHE: Dihydroethidium; RFU: Relative fluorescence units; GPX1: Glutathione peroxidase 1; MPO: Myeloperoxidase; SOD: Superoxide dismutase.

To explore whether the RYGB surgery resulted in amelioration of the oxidative stress status, we analysed several parameters in leukocytes and serum from patients in our study population before and after the intervention (Figure 1).

We observed a diminished leukocyte superoxide production (*p* < 0.05, Figure 1A) and upregulation of the protein expression of the antioxidant enzyme GPX1 (*p* < 0.05, Figure 1B) after the gastric bypass. Moreover, these intracellular changes were accompanied by a drop in systemic levels of MPO (*p* < 0.05, Figure 1C) and an increase in antioxidant SOD activity (*p* < 0.01, Figure 1D), resulting in a significant reduction of the number of carbonyl groups in serum proteins (*p* < 0.01, Figure 1E). Altogether, these results suggest a partial recovery of the redox balance supported by a decrease in prooxidant signalling in favour of antioxidant responses in both leukocytes and serum.

### 3.2. Leukocyte-Endothelial Cell Interactions and CAMs

**Figure 2:**
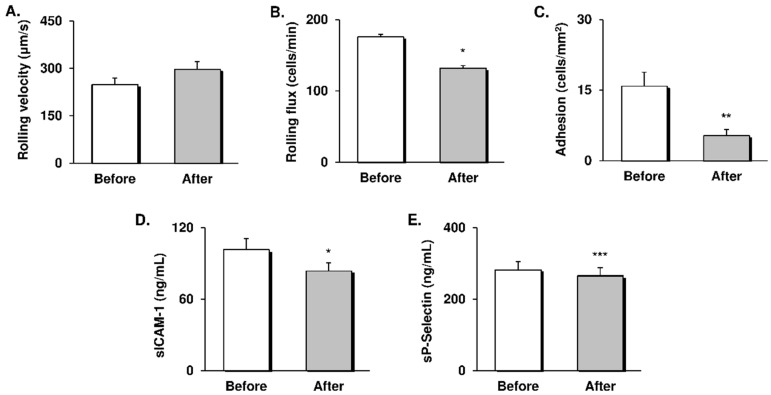
Evaluation of leukocyte-endothelial cell interactions and serum levels of CAMs in obese patients before and 12 months after RYGB intervention. (**A**) Rolling flux, measured as cells per minute (*n* = 24). (**B**) Leukocyte rolling velocity, expressed as µm/s (*n* = 24). (**C**) Leukocyte adhesion, expressed as cells/mm^2^ (*n* = 24). Serum levels of (**D**) sICAM-1 (*n* = 46) and (**E**) sP-selectin (*n* = 46). Data are represented as the mean + SE. * *p* < 0.05, ** *p* < 0.01, *** *p* < 0.001 when compared using a paired Student’s t-test. CAMs: Cellular adhesion molecules; RYGB: Roux-en-Y gastric bypass; sICAM-1: Soluble intracellular adhesion molecule; sP-selectin: Soluble platelet selectin.

To address the effect of RYGB-induced weight loss on leukocyte activation and endothelial dysfunction, we analysed leukocyte-endothelial cell interactions and the levels of CAMs released into the serum (Figure 2).

Although there were no changes in leukocyte rolling velocity (Figure 2A), we did notice a significant decrease in the number of leukocytes rolling along (*p* < 0.05, Figure 2B) and adhering to the endothelium (*p* < 0.01, Figure 2C) one year after the intervention. Accordingly, we observed a significant drop of sICAM-1 (*p* < 0.05, Figure 2D) and sP-selectin (*p* < 0.001, Figure 2E) serum levels. These data suggest that the weight loss induced by RYGB reduced the interactions between leukocytes and the vascular wall and diminished endothelial dysfunction.

### 3.3. LDL and HDL Subfractions

**Figure 3:**
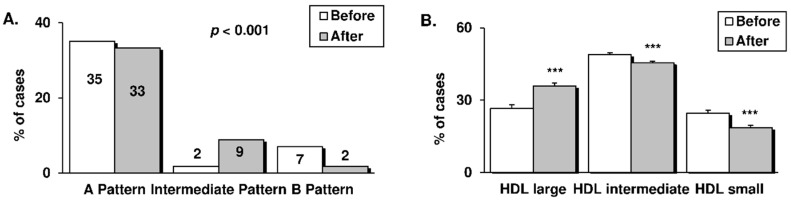
Cholesterol subfractions in obese patients before and 12 months after RYGB intervention, determined by the Quantimetrix Lipoprint^®^ system. (**A**) LDL electrophoretic profiles are expressed as the percentage of patients (*n* = 25) displaying a particular LDL pattern. Patterns refer to the size of LDL particles, as follows: Pattern A (cut-off diameter ≥ 268 Å); Intermediate Pattern (cut-off diameter 264–267 Å) and Pattern B (cut-off diameter ≤ 265 Å). (**B**) HDL profile, expressed as the percentage of patients (*n* = 25) displaying a particular HDL pattern: Large, intermediate, or small. Data are represented as a percentage of LDL patterns or mean + SE. *** *p* < 0.001 when proportions were compared using a X^2^ test or a paired Student’s *t*-test. RYGB: Roux-en-Y gastric bypass; LDL: Low-density lipoprotein; HDL: High-density lipoprotein.

To better understand the modifications in our subjects’ clinical lipid profile after the RYGB intervention we performed a more profound analysis of the circulating cholesterol subfractions (Figure 3).

Beyond the reduction of LDLc levels after the intervention (see Table 1), the evaluation of LDL patterns showed a substantial decrease in the percentage of the more atherogenic sdLDL particles (Pattern B), and an increase in the less atherogenic particles (Intermediate Pattern) (*p* < 0.001), with no significant changes observed with respect to the large LDL particles (Pattern A) (Figure 3A). Further analysis of HDL subfractions revealed a significant increase in the percentage of the considerably antiatherogenic large HDL particles at the expense of a reduction in that of intermediate and small particles (*p* < 0.001, Figure 3B). These observations indicate that patients benefit not only from a quantitative change in cholesterol levels after an RYGB, but also from a parallel qualitative improvement of circulating LDL and HDL subfractions, thus reflecting a less proatherogenic profile.

## 4. Discussion

In our cohort of middle-aged obese subjects, RYGB surgery induced a substantial weight loss one year after the intervention, and this was accompanied by improvements in blood pressure, glycaemic control, inflammation, and lipoprotein particles profile. Beyond these clinical changes, the patients also exhibited a shift from a prooxidant status by which their antioxidant mechanisms were bolstered. In this sense, we observed a drop in superoxide production within leukocytes and systemic MPO levels, while expression of the antioxidant GPX1 enzyme and SOD activity were increased, resulting in lower serum carbonylated proteins. In parallel, we observed an improvement of endothelial function, manifested by a reduction of sICAM-1 and sP-selectin levels and fewer interactions of leukocytes with the vascular wall. As a whole, the present results provide novel and valuable evidence about the molecular mechanisms underlying the protective effects of bariatric surgery against cardiovascular risk and development of atherosclerosis.

Endemic rates of obesity worldwide have fuelled efforts to develop weight loss strategies. Metabolic surgery procedures, including RYGB, have been shown to accomplish, not only weight loss targets, but also recovery from metabolic comorbidities, including the incidence of cardiovascular events. As expected, our patients benefitted from substantial EWL, together with a decrease in BMI and abdominal circumference, reduced blood pressure and IR, and significant improvements in lipid profile one year after the intervention. These gains were reflected in remission rates for hypertension, T2D, and hyperlipidemia of 57.1%, 88.2%, and 61.5%, respectively, which are higher than those reported by other studies [21] and represent a diminished risk of CVD in our obese population.

Atherogenic dyslipidemia is a major contributor to the increased cardiovascular risk currently seen in the general population [22]; it results from a combination of elevated levels of TG and highly atherogenic sdLDL particles together with a decrease in circulating antiatherogenic HDL molecules. As we and other groups have shown, patients after RYGB surgery benefit, not only from quantitative reduction of clinical LDLc and TG levels and an increase in HDLc [23,24,25], but from a complementary qualitative improvement of lipoprotein particles, including a reduction in the percentage of sdLDL and an increased presence of larger HDL [26], which frequently display inverse relationships with cardiovascular risk in epidemiological studies [27]. Furthermore, elevated levels of circulating inflammatory cytokines, and particularly hsCRP, are considered independent predictors of cardiovascular events, even more so than LDLc levels [28]. Indeed, hsCRP is known to rise with the degree of adiposity [8] and is directly involved in the development of atherosclerosis through complement system activation and endothelial dysfunction [29]. In the present study, we report a significant decrease in serum levels of hsCRP, C3c, and total leukocyte count after weight loss induced by RYGB, which is in accordance with previous studies [20,23,24]. Such evidence endorses the long-lasting therapeutic value of bariatric surgery against inflammatory and lipid-related cardiovascular risk [25]. However, few studies have explored the modulation of immune cells response after RYGB. We have gone one step further by focusing on leukocytes as key mediators of inflammation and oxidative stress in the early stages of the atherosclerotic process in obesity.

Oxidative stress is a major mechanism linking obesity, endothelial dysfunction, and the development of atherosclerosis. Although adipose tissue is the main contributor to the oxidative imbalance, other sources of ROS have been implicated in the alteration of NO availability and vascular homeostasis, including excess ROS release by the endothelial NADPH enzyme [30], and mitochondrial dysfunction and subsequent overproduction of superoxide in peripheral leukocytes [8,15,16]. Herein, we report a decrease in superoxide production in the leukocytes of obese patients after RYGB that was associated with upregulation of the expression of antioxidant GPX1, a major scavenger of mitochondrial ROS. Only two previous studies have explored changes in the prooxidant state of leukocytes after bariatric surgery. While Roberts et al. reported diminished superoxide production by immune cells under stimulation [31], Monzo-Beltran et al. observed an adaptive antioxidant response of leukocytes after bariatric surgery in terms of higher intracellular GPX, SOD, and catalase activity [32], which is in line with the present findings. Furthermore, the contribution of rising circulating levels of MPO enzyme to the role of immune cells in endothelial dysfunction is also worthy of mention. This prooxidant enzyme, resulting from the degranulation process of neutrophils, participates in the oxidation of LDL particles and the impairment of eNOS function, and has been associated with an increased risk of coronary artery disease [33,34,35]. In line with this, we have recently demonstrated an association between MPO and sdLDL/sP-selectin levels [36]. In the present study, we detected a drop in MPO serum levels one year after the RYGB intervention, which is in line with previous reports [31]. Additionally, our results revealed a strengthening of systemic antioxidant responses one year after RYGB surgery, since patients showed higher activity of serum SOD and a marked drop in the amount of carbonyl groups in circulating proteins, which is considered a clear biomarker of systemic oxidative stress. In line with these findings, some previous studies have reported a reduction in serum indicators of oxidative stress, including carbonyl proteins, lipid peroxidation, and 8-oxo-dG, which has been associated with an increase in SOD and catalase activity after bariatric surgery [20,32,37]. Considered together, the available evidence endorses RYGB surgery an effective strategy to reduce oxidative damage in patients with obesity by modulating systemic prooxidant and antioxidant responses.

It is generally accepted that elevated circulating levels of CAMs (resulting from immune and endothelial cells activation) reveal endothelial dysfunction and are prognostic of CVD [38], since they are involved in the recruitment of leukocytes by the vascular wall. In the present study, downregulation of MPO and systemic and leukocyte oxidative stress was accompanied by a drop in levels of the adhesion molecules sICAM and sP-selectin, resulting in a marked reduction in the number of leukocytes rolling and firmly adhering to the endothelium after the intervention. Accumulating evidence suggests an association between endothelial dysfunction, the prooxidant state of leukocytes, and their adherent phenotype in patients with obesity and T2D [8,15], since excess superoxide release from immune cells triggers vascular permeability and favours their recruitment and migration [30]. In addition, MPO derived from neutrophils promotes their attraction to the endothelium through physical forces [39]. Conversely, targeting excess ROS production within leukocytes seems to reduce their interaction with the endothelium and the extent of the vascular injury produced [40,41]. Hence, it is likely that an attenuation of the prooxidant phenotype of leukocytes after bariatric surgery contributes to the amelioration of endothelial dysfunction and recruitment of leukocytes, thus protecting against the development of atherosclerosis.

To our knowledge, this is the first time that a decrease in leukocyte activation and endothelial dysfunction after bariatric surgery has been demonstrated by means of a dynamic system in which an in vivo blood flow is simulated and interaction with endothelial cells can be visualised. Previous studies investigating classic functional and structural markers of early atherosclerosis, such as flow-mediated dilation and carotid artery intima-media thickness, have provided inconsistent results concerning the effects of bariatric surgery on endothelial function [19,42]. Indeed, the mechanisms involved in the early asymptomatic stages of atherosclerosis can be triggered many years before clinical signs are manifested [7], thus suggesting that a leukocyte-endothelial cell interaction-based approach would be useful for early detention of subclinical atherosclerosis risk. In this sense, our findings provide more reliable and valuable data on immune response modulation, contributing to a better understanding of the mechanisms underlying the protective effect of bariatric surgery on CVD and atherosclerosis. However, the present study has some limitations, including the relatively small size of the study population, though we would like to point out that it was supported by a sample size calculation. In addition, modifications of functional endothelial markers or the atherosclerotic plaque have not been assessed; however, as commented on above, the evaluation of leukocyte-endothelial cell interactions would allow the early detection of changes in the atherosclerotic process. On the other hand, the scale of the changes studied after RYGB would be more precisely defined by comparison with a control healthy group. Finally, the effect of bariatric surgery on subclinical atherosclerotic mechanisms from a gender perspective is an aspect yet to be explored.

## 5. Conclusions

To conclude, bariatric surgery is an effective strategy for body weight reduction and recovery from metabolic diseases in obese subjects. In addition to confirming these clinical effects, we go a step further by describing the effects of surgically-induced weight loss on the underlying mechanisms of atherosclerosis, including oxidative stress, leukocyte activation, and recruitment to the vessel wall, and atherogenic lipid profile. These novel findings endorse the therapeutic value of bariatric surgery as a way of reducing cardiovascular risk in an obese population.

## Figures and Tables

**Table 1:** Anthropometric and biochemical parameters of the cohort population before and after Roux en-Y gastric bypass (RYGB) intervention.

Parameters	Before	After
*n* (females %)	57 (84.5)	
Age (years)	45.39 ± 10.49	
Weight (kg)	109.3 ± 16.1	78.6 ± 13.0 ***
BMI (kg/m^2^)	39.8 ± 5.3	28.9 ± 4.3 ***
Waist (cm)	115.5 ± 11.2	89.0 ± 11.8 ***
EWL (%)	-	80.4 ± 29.0
SBP (mmHg)	131.8 ± 15.9	122.4 ± 17.6 ***
DBP (mmHg)	81.3 ± 10.3	74.1 ± 10.6 **
Glucose (mg/dL)	98.6 ± 23.6	85.8 ± 11.4 ***
Insulin (μU/mL)	14.7 ± 7.6	6.9 ± 3.0 ***
HOMA-IR	3.8 ± 3.2	1.45 ± 0.7 ***
HbA1c (%)	5.5 ± 0.7	5.2 ± 0.4 ***
TC (mg/dL)	187.7 ± 34.5	166.6 ± 26.4 ***
HDLc (mg/dL)	46.5 ± 8.8	55.0 ± 9.6 ***
LDLc (mg/dL)	122.0 ± 39.7	95.9 ± 21.0 ***
TG (mg/dL)	98.5 (77, 144)	75 (55, 100) ***
hsCRP (mg/L)	3.7 (2.0, 5.5)	0.6 (0.2, 1.2) ***
C3c (mg/L)	126.7 ± 22.9	95.6 ± 17.6 ***
Leukocytes (cells × 10^3^/μL)	7.6 ± 2.3	6.3 ± 1.9 ***
Treatment		
Hypertension % (*n*)	37 (21)	16 (9)
Hyperlipidemia % (*n*)	23 (13)	9 (5)
T2D % (*n*)	30 (17)	4 (2)

Data are expressed as the mean ± SD or percentage (*n*). TG and hsCRP are represented as the median and IQ range (25% and 75% percentile). Values were statistically compared with a paired Student’s *t*-test or Wilcoxon test and were considered significant when ** *p* < 0.01, and *** *p* < 0.001. BMI: Body mass index; EWL: Excess weight loss; SBP: Systolic blood pressure; DBP: Diastolic blood pressure; HOMA-IR: Homeostatic Model Assessment for Insulin Resistance index; HbA1c: Glycated haemoglobin; TC: Total cholesterol; LDLc: LDL cholesterol; HDLc: HDL cholesterol; TG: Triglycerides; hsCRP: High sensitive C-reactive protein; C3c: Complement component 3; T2D: Type 2 diabetes.

## References

[1] Ferrante A.W. (2007). Obesity-induced inflammation: A metabolic dialogue in the language of inflammation. J. Intern. Med..

[2] Grundy S.M. (2004). Obesity, metabolic syndrome, and cardiovascular disease. J. Clin. Endocrinol. Metab..

[3] Whitlock G., Lewington S., Sherliker P., Clarke R., Emberson J., Halsey J., Qizilbash N., Collins R., Peto R., Prospective Studies Collaboration (2009). Body-mass index and cause-specific mortality in 900,000 adults: Collaborative analyses of 57 prospective studies. Lancet.

[4] Despres J.P. (2012). Body fat distribution and risk of cardiovascular disease: An update. Circulation.

[5] Guzik T.J., Mangalat D., Korbut R. (2006). Adipocytokines—Novel link between inflammation and vascular function?. J. Physiol. Pharmacol..

[6] Fontana L., Eagon J.C., Trujillo M.E., Scherer P.E., Klein S. (2007). Visceral fat adipokine secretion is associated with systemic inflammation in obese humans. Diabetes.

[7] Reho J.J., Rahmouni K. (2017). Oxidative and inflammatory signals in obesity-associated vascular abnormalities. Clin. Sci..

[8] Lopez-Domenech S., Bañuls C., Diaz-Morales N., Escribano-Lopez I., Morillas C., Veses S., Orden S., Alvarez A., Victor V.M., Hernandez-Mijares A. (2018). Obesity impairs leukocyte-endothelium cell interactions and oxidative stress in humans. Eur. J. Clin. Investig..

[9] Chattopadhyay M., Khemka V.K., Chatterjee G., Ganguly A., Mukhopadhyay S., Chakrabarti S. (2015). Enhanced ROS production and oxidative damage in subcutaneous white adipose tissue mitochondria in obese and type 2 diabetes subjects. Mol. Cell. Biochem..

[10] Olusi S.O. (2002). Obesity is an independent risk factor for plasma lipid peroxidation and depletion of erythrocyte cytoprotectic enzymes in humans. Int. J. Obes. Relat. Metab. Disord..

[11] Ozata M., Mergen M., Oktenli C., Aydin A., Sanisoglu S.Y., Bolu E., Yilmaz M.I., Sayal A., Isimer A., Ozdemir I.C. (2002). Increased oxidative stress and hypozincemia in male obesity. Clin. Biochem..

[12] Wu M.Y., Li C.J., Hou M.F., Chu P.Y. (2017). New Insights into the Role of Inflammation in the Pathogenesis of Atherosclerosis. Int. J. Mol. Sci..

[13] Nguyen M.T., Fernando S., Schwarz N., Tan J.T., Bursill C.A., Psaltis P.J. (2019). Inflammation as a Therapeutic Target in Atherosclerosis. J. Clin. Med..

[14] Ghanim H., Aljada A., Hofmeyer D., Syed T., Mohanty P., Dandona P. (2004). Circulating mononuclear cells in the obese are in a proinflammatory state. Circulation.

[15] Hernandez-Mijares A., Rocha M., Rovira-Llopis S., Banuls C., Bellod L., de Pablo C., Alvarez A., Roldan-Torres I., Sola-Izquierdo E., Victor V.M. (2013). Human leukocyte/endothelial cell interactions and mitochondrial dysfunction in type 2 diabetic patients and their association with silent myocardial ischemia. Diabetes Care.

[16] Bañuls C., Rovira-Llopis S., Marañon A.M.d., Veses S., Jover A., Gomez M., Rocha M., Hernandez-Mijares A., Victor V.M. (2017). Metabolic syndrome enhances endoplasmic reticulum, oxidative stress and leukocyte-endothelium interactions in PCOS. Metab. Clin. Exp..

[17] Ikramuddin S., Korner J., Lee W.J., Connett J.E., Inabnet W.B., Billington C.J., Thomas A.J., Leslie D.B., Chong K., Jeffery R.W. (2013). Roux-en-Y gastric bypass vs intensive medical management for the control of type 2 diabetes, hypertension, and hyperlipidemia: The Diabetes Surgery Study randomized clinical trial. JAMA.

[18] Sjostrom L., Peltonen M., Jacobson P., Sjostrom C.D., Karason K., Wedel H., Ahlin S., Anveden A., Bengtsson C., Bergmark G. (2012). Bariatric surgery and long-term cardiovascular events. JAMA.

[19] Lupoli R., Di Minno M.N., Guidone C., Cefalo C., Capaldo B., Riccardi G., Mingrone G. (2016). Effects of bariatric surgery on markers of subclinical atherosclerosis and endothelial function: A meta-analysis of literature studies. Int. J. Obes..

[20] Joao Cabrera E., Valezi A.C., Delfino V.D., Lavado E.L., Barbosa D.S. (2010). Reduction in plasma levels of inflammatory and oxidative stress indicators after Roux-en-Y gastric bypass. Obes. Surg..

[21] Puzziferri N., Roshek T.B., Mayo H.G., Gallagher R., Belle S.H., Livingston E.H. (2014). Long-term follow-up after bariatric surgery: A systematic review. JAMA.

[22] Franssen R., Monajemi H., Stroes E.S., Kastelein J.J. (2008). Obesity and dyslipidemia. Endocrinol. Metab. Clin. N. Am..

[23] Minervino D., Gumiero D., Nicolazzi M.A., Carnicelli A., Fuorlo M., Guidone C., Di Gennaro L., Fattorossi A., Mingrone G., Landolfi R. (2015). Leukocyte Activation in Obese Patients: Effect of Bariatric Surgery. Medicine.

[24] Gomez-Abril S.A., Morillas-Ariño C., Ponce-Marco J.L., Torres-Sanchez T., Delgado-Gomis F., Hernandez-Mijares A., Rocha M. (2016). Short- and Long-Term Effects of Weight Loss on the Complement Component C3 After Laparoscopic Gastric Bypass in Obese Patients. Obes. Surg..

[25] Al-Zoairy R., Melmer A., Ress C., Laimer M., Kaser S., Ebenbichler C. (2012). Lipid profile changes after pronounced weight loss induced by bariatric surgery. Clin. Lipidol..

[26] Kjellmo C.A., Karlsson H., Nestvold T.K., Ljunggren S., Cederbrant K., Marcusson-Stahl M., Mathisen M., Lappegard K.T., Hovland A. (2018). Bariatric surgery improves lipoprotein profile in morbidly obese patients by reducing LDL cholesterol, apoB, and SAA/PON1 ratio, increasing HDL cholesterol, but has no effect on cholesterol efflux capacity. J. Clin. Lipidol..

[27] Kontush A. (2015). HDL particle number and size as predictors of cardiovascular disease. Front. Pharmacol..

[28] Ridker P.M., Rifai N., Rose L., Buring J.E., Cook N.R. (2002). Comparison of C-reactive protein and low-density lipoprotein cholesterol levels in the prediction of first cardiovascular events. N. Engl. J. Med..

[29] Badimon L., Pena E., Arderiu G., Padro T., Slevin M., Vilahur G., Chiva-Blanch G. (2018). C-Reactive Protein in Atherothrombosis and Angiogenesis. Front. Immunol..

[30] Csanyi G., Taylor W.R., Pagano P.J. (2009). NOX and inflammation in the vascular adventitia. Free Radic. Biol. Med..

[31] Roberts H.M., Grant M.M., Hubber N., Super P., Singhal R., Chapple I.L.C. (2018). Impact of Bariatric Surgical Intervention on Peripheral Blood Neutrophil (PBN) Function in Obesity. Obes. Surg..

[32] Monzo-Beltran L., Vazquez-Tarragon A., Cerda C., Garcia-Perez P., Iradi A., Sanchez C., Climent B., Tormos C., Vazquez-Prado A., Girbes J. (2017). One-year follow-up of clinical, metabolic and oxidative stress profile of morbid obese patients after laparoscopic sleeve gastrectomy. 8-oxo-dG as a clinical marker. Redox Biol..

[33] Carr A.C., McCall M.R., Frei B. (2000). Oxidation of LDL by myeloperoxidase and reactive nitrogen species: Reaction pathways and antioxidant protection. Arterioscler. Thromb. Vasc Biol..

[34] Brennan M.L., Hazen S.L. (2003). Emerging role of myeloperoxidase and oxidant stress markers in cardiovascular risk assessment. Curr. Opin. Lipidol..

[35] Lau D., Baldus S. (2006). Myeloperoxidase and its contributory role in inflammatory vascular disease. Pharmacol. Ther..

[36] Lopez-Domenech S., Martinez-Herrera M., Abad-Jimenez Z., Morillas C., Escribano-Lopez I., Diaz-Morales N., Banuls C., Victor V.M., Rocha M. (2019). Dietary weight loss intervention improves subclinical atherosclerosis and oxidative stress markers in leukocytes of obese humans. Int. J. Obes (Lond).

[37] Da Silva V.R., Moreira E.A., Wilhelm-Filho D., de Miranda J.X., Beninca J.P., Vigil S.V., Moratelli A.M., Garlet T.R., de Souza Meirelles M.S., Vannucchi H. (2012). Proinflammatory and oxidative stress markers in patients submitted to Roux-en-Y gastric bypass after 1 year of follow-up. Eur. J. Clin. Nutr..

[38] Bielinski S.J., Berardi C., Decker P.A., Kirsch P.S., Larson N.B., Pankow J.S., Sale M., de Andrade M., Sicotte H., Tang W. (2015). P-selectin and subclinical and clinical atherosclerosis: The Multi-Ethnic Study of Atherosclerosis (MESA). Atherosclerosis.

[39] Klinke A., Nussbaum C., Kubala L., Friedrichs K., Rudolph T.K., Rudolph V., Paust H.J., Schroder C., Benten D., Lau D. (2011). Myeloperoxidase attracts neutrophils by physical forces. Blood.

[40] Garg R., Kumbkarni Y., Aljada A., Mohanty P., Ghanim H., Hamouda W., Dandona P. (2000). Troglitazone reduces reactive oxygen species generation by leukocytes and lipid peroxidation and improves flow-mediated vasodilatation in obese subjects. Hypertension.

[41] Escribano-Lopez I., Diaz-Morales N., Iannantuoni F., Lopez-Domenech S., de Maranon A.M., Abad-Jimenez Z., Banuls C., Rovira-Llopis S., Herance J.R., Rocha M. (2018). The mitochondrial antioxidant SS-31 increases SIRT1 levels and ameliorates inflammation, oxidative stress and leukocyte-endothelium interactions in type 2 diabetes. Sci. Rep..

[42] Borzi A.M., Buscemi C., Corleo D., Randazzo C., Rosafio G., Pantuso G., Buscemi S. (2020). Endothelial Function in Obese Patients Treated with Bariatric Surgery. Diabetes Metab. Syndr. Obes.

